# Trivalent Y^3+^ ionic sensor development based on (*E*)-Methyl-*N*′-nitrobenzylidene-benzenesulfonohydrazide (MNBBSH) derivatives modified with nafion matrix

**DOI:** 10.1038/s41598-017-05703-4

**Published:** 2017-07-19

**Authors:** Mohammad Musarraf Hussain, Mohammed M. Rahman, Muhammad Nadeem Arshad, Abdullah M. Asiri

**Affiliations:** Chemistry Department and Center of Excellence for Advanced Material Research, Faculty of Science, King Abdulaziz University, Jeddah, 21589 P.O. Box 80203, Saudi Arabia

## Abstract

(*E*)-Methyl-*N*′-nitrobenzylidene-benzenesulfonohydrazide (MNBBSH) compounds were synthesized using a condensation procedure from the derivatives of nitrobenzaldehyde and 4-Methyl-benzenesulfonylhydrazine, which crystallized in ethanol and methanol as well as characterized by FTIR, UV-Vis, ^1^H-NMR, and ^13^C-NMR. MNBBSH structure was confirmed using a single crystal X-ray diffraction technique and used for the detection of selective yttrium ion (Y^3+^) by I-V system. A thin layer of MNBBSH was deposited onto a glassy carbon electrode (GCE) with 5% nafion for the sensitive and selective Y^3+^ sensor. The modified MNBBSH/GCE sensor is exhibited the better electrochemical performances such as sensitivity, limit of detection (LOD), linear dynamic range (LDR), limit of quantification (LOQ), short response time, and long term storage ability towards the selective metal ion (Y^3+^). The calibration curve of 2-MNBBSH/GCE sensor was plotted at +1.1 V over a broad range of Y^3+^ concentration. Sensitivity, LOD, LDR and LOQ of the fabricated sensor towards Y^3+^ were calculated from the calibration curve and found as 1.90 pAμM^−1^ cm^−2^, 10.0 pM, 1.0 nM~1.0 mM and 338.33 mM respectively. The 2-MNBBSH/Nafion/GCE sensor was applied to the selective determination of Y^3+^ in spiked samples such as industrial effluent and real water samples from different sources, and found acceptable and reasonable results.

## Introduction

Organic molecules having electron donor functional group is an immense attention in chemistry due to enormous number of applications in biological, catalysis, environmental, medicinal, and organometallic area by binding of diverse metal constituents^1–4^. Sulfonamides are acknowledged as sulfa drugs, and used as antibiotics in bacterial category infections in animals, and human beings. Sulfa drugs are also applied in the treatment of bacillary dysentery, conjunctivitis, eye & gut infections, malaria, meningitis, and urinary tract infections^5, 6^. Yttrium tungstate [Y_2_(WO_4_)_3_, orthorhombic structure] shows large negative thermal expansion beside all the three crystallographic axes^7^. Y-90 is a chaste *β* particle emitting radionuclide which is appropriate for the application in nuclear medicine such as radiolabeling of pharmaceuticals for bone palliation, cancer treatment, radiation synovectomy, rheumatoid arthritis, and tumor therapy. Several techniques for example extraction chromatography, ion exchange, precipitation, and solvent extraction used to purify and separate of Y-90^8, 9^.

Sensors based on alkyl oxymercuration, DNA, oligonucleotide, protein, and little organic compounds have been developed and experienced for heavy metal ions (HMI). However, these techniques are inconvenience for Y^3+^ exposure in biological and environmental samples due to numerous limitations such as high functioning temperatures, lower-water solubility, nitrogen-purge buffer, time consuming, and too expensive. Hence it is necessary to build up an easy, sensitive, and dependable method for the detection of toxic metallic ions for environmental security, food quality control, protection, and human being health arena^10^. Electrochemical sensing of poisonous molecules represents a proficient advancement that can be used to harmonize earlier available techniques owing to combine features such as simple instrumentation, little cost, extremely selective and sensitive, and considerable for effectiveness in order to detect HMI^11, 12^. In this research work, an efficient sensor has been developed in order to detect the trivalent metal ion (Y^3+^) using (*E*)-Methyl-*N*′-nitrobenzylidene-benzenesulfonohydrazide derivatives. To best of our knowledge, it is the first report for selective and sensitive identification and determination of Y^3+^ using 2-MNBBSH by a dependable I-V performance with short response time.

## Experimental

### Material and methods

The chemicals of analytical ranking for example 2-Nitrobenzaldehyde, 3-Nitrobenzaldehyde, 4-Nitrobenzaldehyde, 4-Methyl-benzenesulfonylhydrazine, AgNO_3_, AuCl_3_, CdSO_4_, Co(NO_3_)_2_, Cr(NO_3_)_3_, HgCl_2_, NiCl_2_, Y(NO_3_)_3_, ZnSO_4_, EtOH, MeOH, NaH_2_PO_4_, Na_2_HPO_4_, and nafion (5% ethanolic solution) were purchased from the Sigma Aldrich company, and used as received. A mother solution of Y^3+^ (100.0 mM) was prepared from the purchased chemical, Y(NO_3_)_3_. A Stuart scientific SMP3 (version 5.0) melting point apparatus (Bibby Scientific Limited, Staffordshire, UK) was used to record the melting point (m.p.), and the reported m.p. was not corrected. ^1^H-NMR, and ^13^C-NMR spectra were recorded on an AVANCE-III instrument (400 MHz, Bruker, Fallanden, Switzerland) at 300 k, and chemical shifts were reported in ppm with reference with residual solvent signal. FTIR spectra were recorded as neat on a Thermo scientific NICOLET iS50 FTIR spectrometer (Madison, WI, USA). UV-V is study was conducted using Evolution 300 UV-Vis spectrophotometer (Thermo scientific). I-V method was carried out in order to detect Y^3+^ at a selective point using the modified 2-MNBBSH/GCE by Keithley electrometer (6517A, USA). Warning! Yttrium is poisonous. So, a minute amount of this chemical can be used for the preparation of the essential solution with care.

### Synthesis of MNBBSH derivatives

#### (E)-4-Methyl-N′-(2-nitrobenzylidene)-benzenesulfonohydrazide (2-MNBBSH, 3)

A mixture of 2-nitrobenzaldehyde (514.3 mg, 3.38 mmol, 1.22 equiv), and 4-methyl-benzenesulfonylhydrazine (513.6 mg, 2.76 mmol, 1 equiv) in EtOH (30.0 mL) was stirred at room temperature (R.T.) for 3.30 h. Filtered, and the solution was then kept at open air to evaporate the solvent. The obtained product was crystallized from EtOH to give the title compound **3** as a yellow crystal (757.0 mg, 86%). EF = C_14_H_13_N_3_O_4_S, MW = 319.04, EA = C: 52.66, H: 4.10, N: 13.16, O: 20.04, S: 10.04. m. p. = 160.5–165.2 °C. ^**1**^ **H-NMR** (400 MHz, DMSO-*d*_6_), δ: 11.91 (s, 1H), 8.31 (s, 1H), 8.03 (dd, *J* = 8.2, 1.2 Hz, 1 H), 7.84 (dd, *J* = 7.9, 1.5 Hz, 1H), 7.81–7.73 (m, 3H), 7.64 (ddd, *J* = 8.7, 7.4, 1.5 Hz, 1H), 7.44 (d, *J* = 8.0 Hz, 2H), 2.38 (s, 3H). ^**13**^ **C-NMR** (101 MHz, DMSO-*d*_6_), δ: 189.82, 147.82, 143.64, 142.27, 136.06, 133.69, 130.66, 129.81, 129.75, 127.95, 127.81, 127.14, 124.59, 20.98. **FTIR** (neat) *v*_max_: 3200, 1900, 1805, 1710, 1605, 1470, 1335, 1195, 1085, 915, 845, 800, 725, 685, 535. **UV-Vis** (*λ*_max_): 287.5 nm.

#### (E)-4-Methyl-N′-(3-nitrobenzylidene)-benzenesulfonohydrazide (3-MNBBSH, 4)

A mixture of 3-nitrobenzaldehyde (513.3 mg, 3.37 mmol, 1.21 equiv), and 4-methyl-benzenesulfonylhydrazine (517.6 mg, 2.78 mmol, 1 equiv) in EtOH (30.0 mL) was stirred at R.T. for 2.30 h. Filtered, and subsequently the solution was kept at open air to evaporate the solvent. The found product was crystallized from EtOH to give the title compound **4** as a yellowish crystal (1.05 g, 97%). EF = C_14_H_13_N_3_O_4_S, MW = 319.34, EA = C: 52.66, H: 4.10, N: 13.16, O: 20.04, S: 10.04. m. p.0 = 166.2–179.8 °C. ^**1**^ **H-NMR** (400 MHz, DMSO-*d*_6_), δ: 11.80 (s, 1H), 8.37 (t, *J* = 2.0 Hz, 1H), 8.23 (ddt, *J* = 8.2, 2.2, 1.0 Hz, 1H), 8.09–7.98 (m, 2H), 7.83–7.76 (m, 2H), 7.70 (t, *J* = 7.9 Hz, 1H), 7.43 (d, *J* = 8.0 Hz, 2H), 2.38 (s, 3H). ^**13**^ **C-NMR** (101 MHz, DMSO-*d*_6_), δ: 148.14, 144.55, 143.63, 136.02, 135.44, 132.63, 130.39, 129.73, 127.15, 124.27, 121.00, 20.97. **FTIR** (neat) *v*_max_: 3200, 1905, 1807, 1705, 1610, 1485, 1330, 1265, 1193, 1090, 910, 855, 805, 720, 695, 540. **UV-Vis**. (*λ*_max_): 294.0 nm.

#### (E)-4-Methyl-N′-(4-nitrobenzylidene)-benzenesulfonohydrazide (4-MNBBSH, 5)

A combination of 4-nitrobenzaldehyde (512.8 mg, 3.37 mmol, 1.23 equiv), and 4-methyl-benzenesulfonylhydrazine (512.0 mg, 2.75 mmol, 1 equiv) in EtOH (25.0 mL) was stirred at R.T. for 3.0 h. Filtered, and MeOH was added with the precipitates. The obtained products were crystallized from MeOH to give the title molecule **5** as a white crystal (752.4 mg, 86%). EF = C_14_H_13_N_3_O_4_S, MW = 319.06 g/mol, EA = C: 52.66, H: 4.10, N: 13.16, O: 20.04, S: 10.04. m. p.0 = 166.3–169.2 °C. ^**1**^ **H-NMR** (400 MHz, DMSO-*d*_6_), δ: 11.89 (s, 1H), 8.28–8.21 (m, 2H), 8.03 (s, 1H), 7.88–7.76 (m, 4H), 7.43 (d, *J* = 8.1 Hz, 2H), 2.38 (s, 3H). ^**13**^ **C-NMR** (101 MHz, DMSO-*d*_6_), δ: 147.84, 144.32, 143.69, 139.80, 135.97, 129.75, 127.66, 127.15, 124.24, 124.00, 20.98. **FTIR** (neat) *v*_max_: 3200, 1910, 1803, 1700, 1615, 1490, 1325, 1255, 1197, 1075, 905, 820, 802, 715, 665, 545. **UV-Vis**. (*λ*_max_): 326.0 nm.

#### Crystallography investigation of MNBBSH molecules

Three novel benzenesulfonylhydrazides (**3–5**) were synthesized and crystallized from EtOH, EtOH, and MeOH respectively at R.T. under slow evaporation method. Extremely good-looking, grains like crystals were found in vials. Samples were screened out under microscope for good crystals to mount on instrument for data collection. The assembly used to mount samples consists of a glass fiber inserted in the wax fixed onto the hollow copper tube supported by magnetic base. The specific samples were glued over glass needle and mounted on agilent super nova (Dual source) diffractometer, equipped with microfocus Cu-Mo Kα radiation. The data collection was accomplished using CrysAlisPro software at 296 K under the Cu Kα radiation^13^. The structures were performed and refined by full–matrix least–squares methods on *F* ^*2*^ by means of SHELXL–97 in-built with WinGX^14^. The non-hydrogen atoms were also refined anisotropically using the same procedure. The crystal structure and hydrogen bonding of single crystals were generated through PLATON and ORTEP in built with WinGX^15–17^. All the C_*aromatic*_-H hydrogen atoms were positioned geometrically and treated as riding atoms with C–H = 0.93 Å and Uiso (H) = 1.2 Ueq (C) carbon atoms. The N-H hydrogen atoms were located through Fourier map and refined with N-H = 0.82 (3) Å with Uiso (H) was set to 1.2 Ueq for N atom. The Crystal data were deposited at the Cambridge crystallographic data centre and following deposition numbers have been assigned 1523454, 1523455 and 1523456 which are known as CCDC number for molecules **3–5** respectively. Crystal data can be received free of charge on application to CCDC 12 Union Road, Cambridge CB21 EZ, UK. (Fax: (+44) 1223 336–033; e-mail: data_request@ccdc.cam. ac.uk).

#### Modification of GCE with MNBBSH molecules

**Figure 1:**
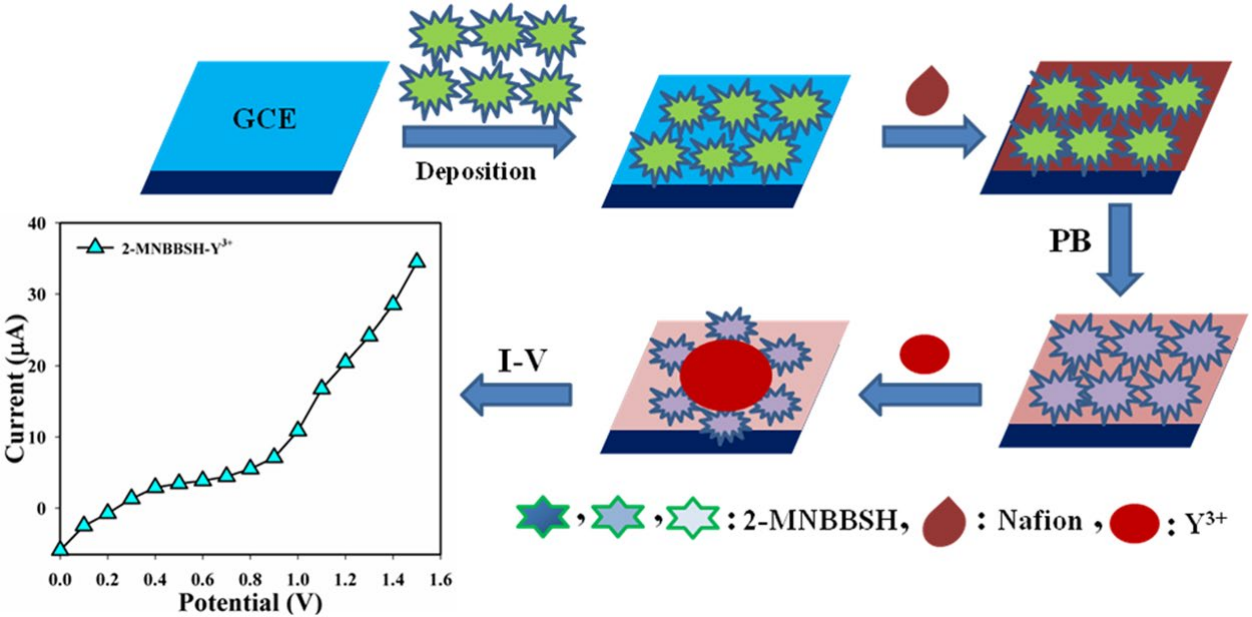
Modification of GCE with MNBBSH derivatives and respective I-V responses.

NaH_2_PO_4_ (93.5, 68.5, 39.0, 16.0, and 5.3 mL), Na_2_HPO_4_ (6.5, 31.5, 61.0, 84.0, and 94.7 mL), and distilled water (500.0 mL) were used for the preparation of a set of phosphate buffer, PB (pH = 5.7, 6.5, 7.0, 7.5, and 8.0). EtOH and nafion (coating binder) was used to modify GCE with MNBBSH compounds. The fabricated electrodes were then kept at R.T. for 3 h in order to development of uniform film with complete drying. The modified GCE and platinum (Pt) wire were used as a working, and counter electrode successively to examine the I-V responses (Fig. 1).

## Results and Discussion

### Spectroscopic studies of MNBBSH molecules

**Figure 2:**
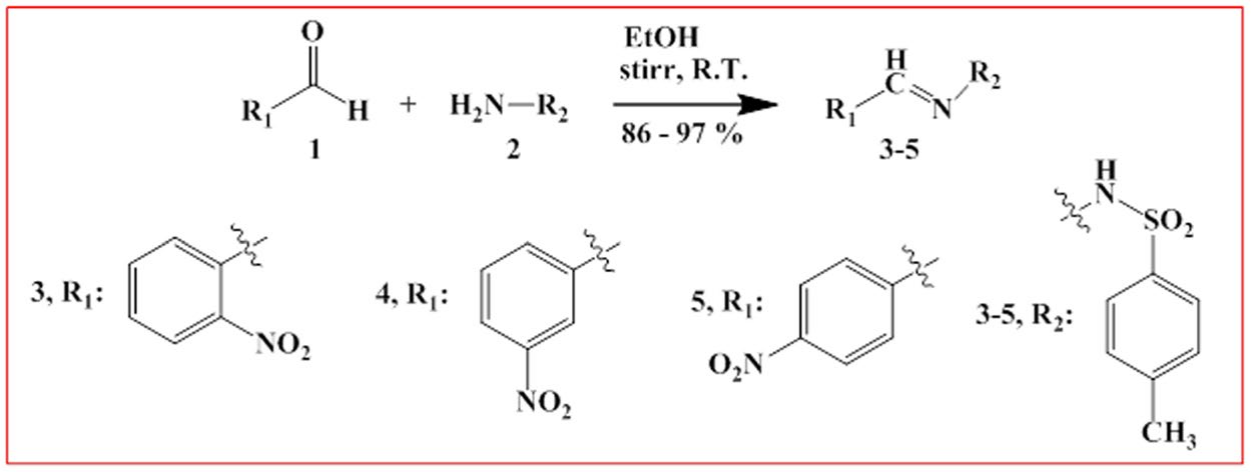
Synthesis of MNBBSH compounds condensation method.

The title compounds (**3–5**) were prepared via a simple condensation method of 2-nitrobenzaldehyde, 3-nitrobenzaldehyde, and 4-nitrobenzaldehyde (**1**), and 4-methyl-benzenesulfonylhydrazine (**2**) with good yield (Fig. 2)^18^. Similar molecules already been reported in the earlier studies^19–26^.

The synthesized molecules **3–5** were characterized using diverse spectroscopic methods, and as a final point the structures were established by means of single crystal X-ray diffraction examination. The purity of compounds gave good spectra which helped us to identify the available proton in the molecules using chemical shift (δ), and coupling constant (*J*) values. One NH proton of the target molecules **3–5** shows singlet at δ 11.91, 11.80, and 11.89 respectively. Both of phenyl groups of the synthesized molecules (**3–5**) have eight protons that appeared in different signals. Aromatic protons found magnificently at their low field region at δ 7.44–8.31, 7.43–8.37, and 7.43–8.28 correspondingly for **3–5**. One singlet observed at δ 2.38 shows three proton and this may be due to CH_3_. ^13^C-NMR spectra were also recorded and most of the carbon atoms was found in the aromatic region (Figs S1–6). FTIR spectroscopy has been examined at 4000–400 cm^−1^ for the structure by defining functional groups *via* different bending, and stretching peaks observed at a particular region in the spectra. UV-Vis spectra was recorded in DMSO quantitatively (200–800 nm), and the λ_max_ were found at 287.5, 294.0, and 326.0 nm which is due to the π-π^*^ transition of the imine functional group (C=NH) in the title molecules **3–5** (Fig. S7–9).

### Crystallography studies of MNBBSH molecules

**Figure 3:**
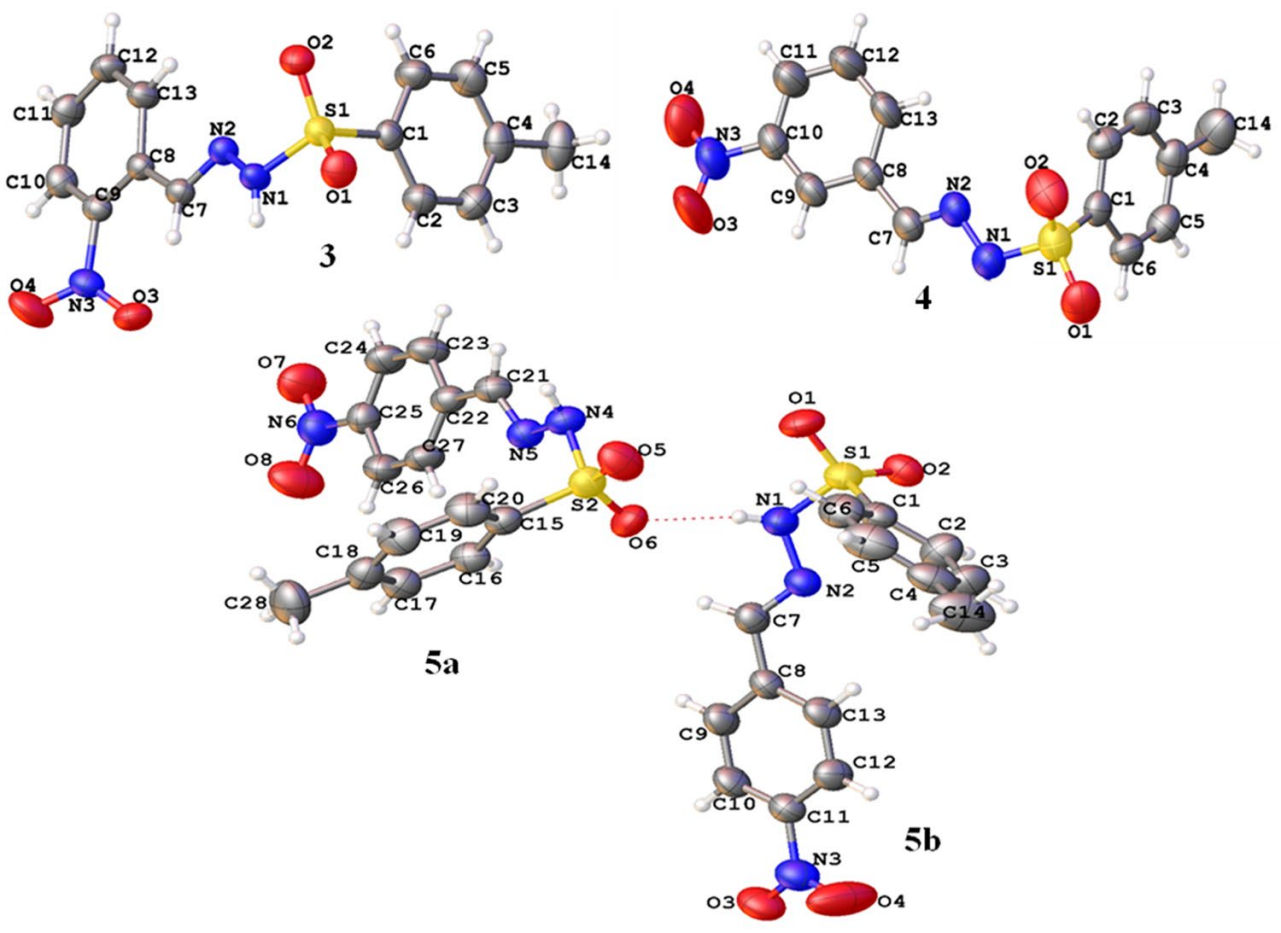
Crystal structure of the MNBBSH molecules.

**Figure 4:**
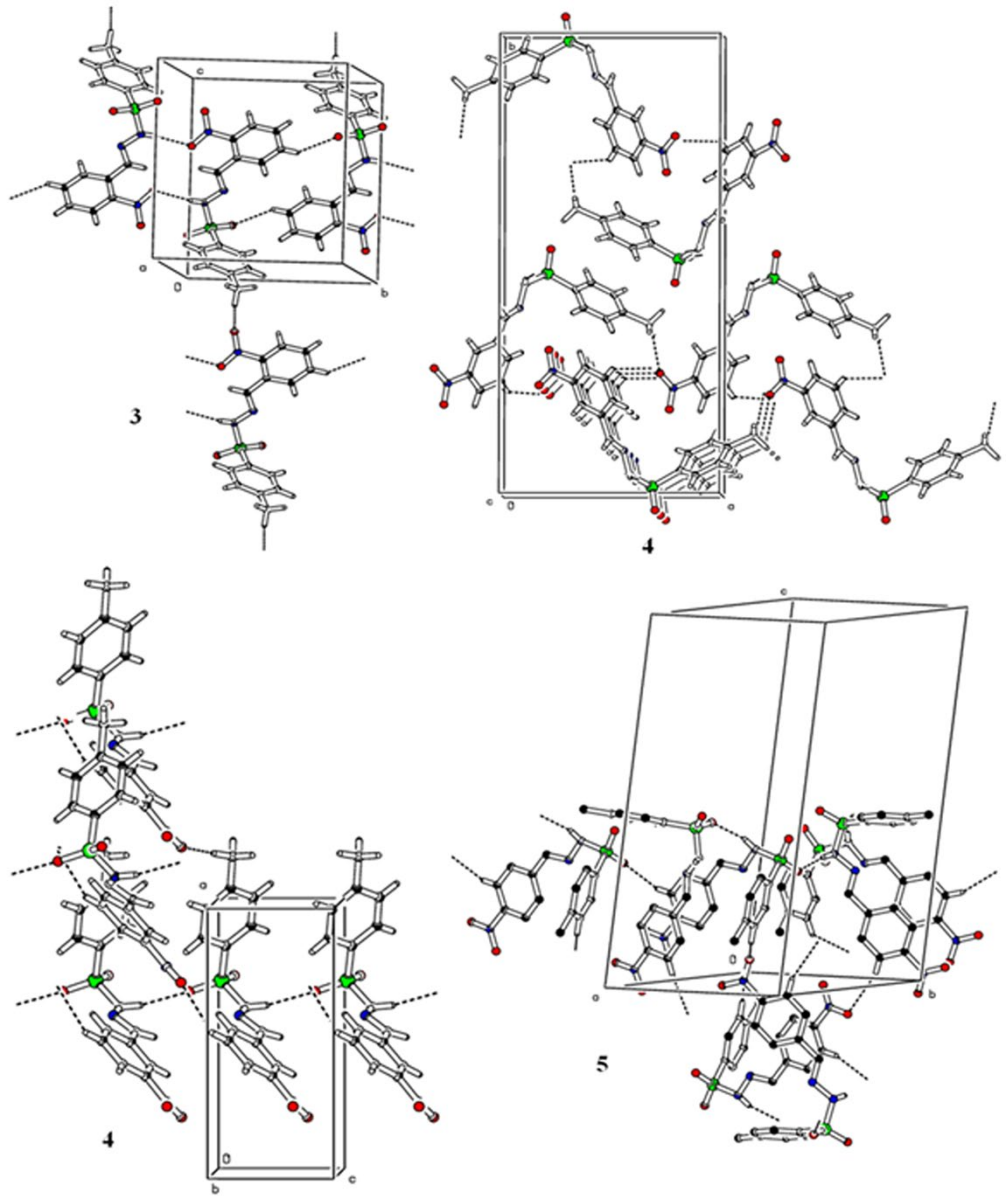
Hydrogen bonding pattern of the MNBBSH compounds.

As we know the structural arrangements of molecules in crystal structure predicts their various properties so we crystallized the synthesized compounds and diffracted them on a single crystal diffractometer. Three (**3–5**) molecules were crystallized in different crystal system. The molecule **3** was crystallized in triclinic crystal system with *P-1* space group, **4** adopted the orthorhombic crystal system with *Pna2* space group and **5** accepted the monoclinic system with *P21/a* space group (Table 1). The compound **5** have two independent molecules in a single asymmetric unit cell. The molecules adopted the V shape having lower corner at S atom in each set. The S atom in the typical sulfonamide functional group adopted distorted tetrahedral geometry with <O1-S1-O2 = 120.43 (1)°, <O1-S1-O2 = 120.14 (2)°, <O1-S1-O2 = 120.08 (1)° and <O1-S1-O2 = 120.13 (1)° for molecules **3–5** respectively^27–30^. The dihedral angles between the two aromatic rings (C1-C6) and (C8-C13) are 86.41 (1)°, 73.12 (2)° in molecule **3**, and **4**. As compound **5** have two independent molecules so the dihedral angles between two aromatic rings are 72.85(2)° and 74.26(1)° in molecules **5a** and **5b** respectively. The nitro groups (NO_2_) are not planar with the aromatic rings to which these are attached and make dihedral angles of 28.86 (2)°, 16.32 (8)°, 6.01 (2)° and 1.93 (1)° for **3**, **4**, **5a**, and **5b** correspondingly (Fig. 3, and Table S1). The molecules also undergo the inter-molecular hydrogen bonding interactions to get them stabilize in their crystal structures. The molecules in crystal structure of **3** followed the classical and non-classical N-H…O and C-H…O interactions which connects them to build a two dimensional network along the plane *bc*. N-H…O interaction from the hydrazide and nitro group connect the molecules to form a sixteen member ring motif *R*_2_^2^(16) [31], while C-H…O interaction generated another eighteen member ring motif. Both of these rings connect to each other to form a polymeric chain along *b* axes. One hydrogen atom from methyl group connects these chains along *c* axes *via* C-H…O interaction. In compound **4**, nitro group involved in non-classical hydrogen bonding interactions where O3 atoms from two different molecules connects the two different hydrogen atoms of same molecule and form long chain which runs across the *a* axes. There is another classical interaction through the hydrazide and SO_2_ group which connects the molecules along *c* axes, and we observed chains of two dimensional networks in molecule **5** which produced through the N-H…O and C-H…O interactions (Fig. 4, Table 2, and Table S2).

**Table 1:** Crystal parameters and structure refinement of the MNBBSH molecules.

Parameters	MNBBSH molecules		
	3	4	5
Identification code	16101	16069	16070
CCDC No	1523454	1523455	1523456
Empirical formula	C_14_H_13_N_3_O_4_S	C_14_H_13_N_3_O_4_S	C_14_H_13_N_3_O_4_S
Formula weight	319.33	319.33	319.33
Temperature/K	296.15	296.15	296.15
Crystal system	triclinic	orthorhombic	monoclinic
Space group	P-1	Pna2_1_	P2_1_/a
a/Å	5.6589(4)	11.4510(12)	15.0244(8)
b/Å	10.5075(8)	25.587(3)	10.2232(5)
c/Å	12.1849(8)	5.1827(5)	20.0576(10)
α/°	93.869(5)	90	90
β/°	95.001(6)	90	102.826(5)
γ/°	98.126(6)	90	90
Volume/Å^3^	712.18(9)	1518.5(3)	3003.9(3)
Z	2	4	8
ρ_calc_mg/mm^3^	1.489	1.397	1.412
µ/mm^-1^	0.250	0.234	0.237
F(000)	332.0	664.0	1328.0
Crystal size/mm^3^	0.43 × 0.28 × 0.12	0.44 × 0.18 × 0.15	0.32 × 0.09 × 0.06
2θ range for data collection	6.736 to 58.866°	5.956 to 58.842°	5.766 to 58.634°
Index ranges	−7 ≤ h ≤ 6, −10 ≤ k ≤ 13, −16 ≤ l ≤ 15	−15 ≤ h ≤ 15, −31 ≤ k ≤ 32, −6 ≤ l ≤ 6	−20 ≤ h ≤ 13, −13 ≤ k ≤ 13, −27 ≤ l ≤ 25
Reflections collected	5390	12184	18281
Independent reflections	3361[R(int) = 0.0160]	3678[R(int) = 0.0279]	7291[R(int) = 0.0380]
Data/restraints/parameters	3361/0/204	3678/1/205	7291/0/407
Goodness-of-fit on F^2^	1.048	1.100	1.027
Final R indexes [I > = 2σ (I)]	R_1_ = 0.0429, wR_2_ = 0.1002	R_1_ = 0.0473, wR_2_ = 0.1072	R_1_ = 0.0553, wR_2_ = 0.1180
Final R indexes [all data]	R_1_ = 0.0559, wR_2_ = 0.1118	R_1_ = 0.0711, wR_2_ = 0.1210	R_1_ = 0.1099, wR_2_ = 0.1415
Largest diff. peak/hole/e Å^−3^	0.28/−0.31	0.14/−0.25	0.24/−0.33
Flack parameter	—	0.08(14)	—

**Table 2:** Hydrogen bonding of the MNBBSH compounds.

MNBBSH	D	H	A	d(D-H)/Å	d(H-A)/Å	d(D-A)/Å	D-H-A/°
**3**	C12	H12	O2^1^	0.93	2.57	3.249(2)	130.5
	C14	H14C	O4^2^	0.96	2.57	3.364(3)	140.5
	N1	H1N	O3^3^	0.82(2)	2.21(2)	3.024(2)	171(2)
	^1^2-X,1-Y,1-Z; ^2^1 + X, +Y,1 + Z; ^3^1-X,2-Y,1-Z						
**4**	N1	H1N	O2^1^	0.88(5)	2.09(6)	2.952(5)	166(4)
	C12	H12	O3^2^	0.93	2.48	3.222(6)	136.9
	C14	H14B	O3^3^	0.96	2.47	3.385(7)	159.8
	^1^ + X, +Y,1 + Z; ^2^1/2 + X,1/2-Y,−1 + Z; ^3^1 + X, +Y,−1 + Z						
**5**	N4	H4N	O2^1^	0.87(3)	2.06(3)	2.906(3)	163(2)
	C10	H10	O7^2^	0.93	2.58	3.465(3)	159.6
	C17	H17	O3^3^	0.93	2.58	3.280(4)	131.9
	C24	H24	O6^4^	0.93	2.44	3.336(3)	160.9
	N1	H1N	O6	0.80(3)	2.13(3)	2.917(3)	171(3)
	^1^1/2 + X,1/2-Y, +Z; ^2^-X,-Y,-Z; ^3^-X,1-Y,-Z; ^4^ + X, −1 + Y, +Z						

## Application

### Detection of trivalent Y^3+^ by 2-MNBBSH using I-V technique

**Figure 5:**
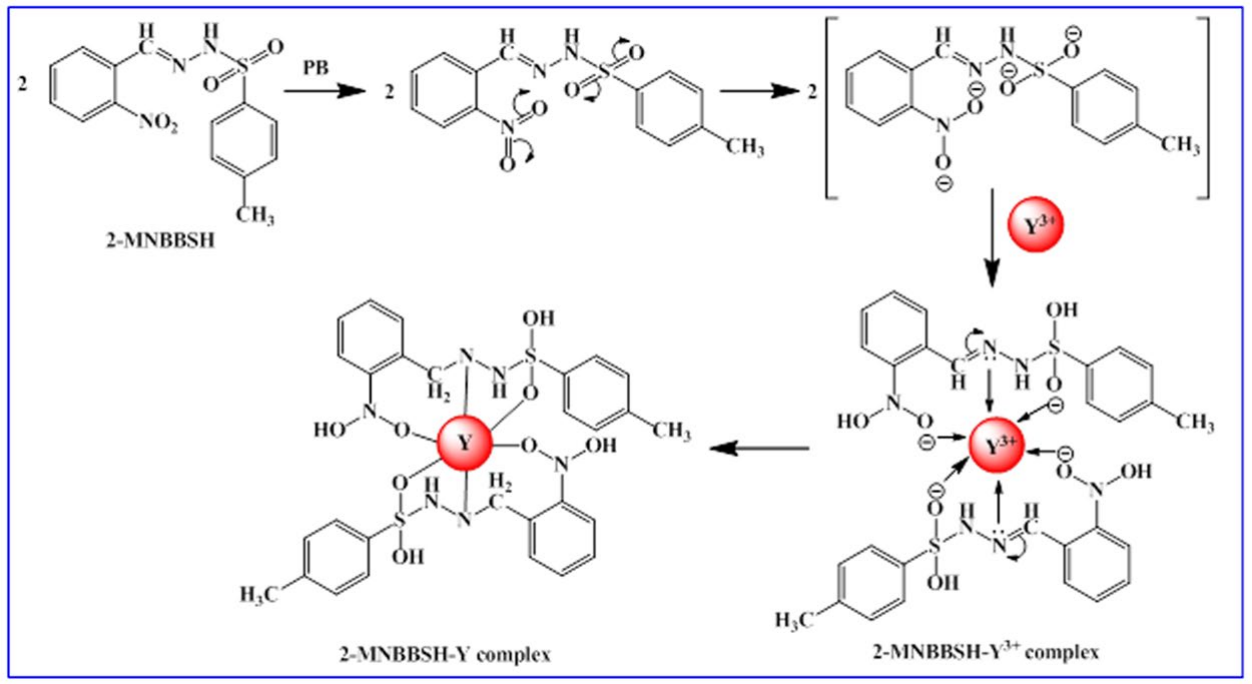
Possible mechanism of 2-MNBBSH-Y-complex formation.

Enhancement of the modified electrode with organic molecule is the beginning stage of application as a metal ion (MI) sensor. The significant use of MNBBSH fabricated onto the GCE was examined as a MI sensor for the detection and quantity of the desired cation, Y^3+^ in PB. The MNBBSH/GCE sensor comprised diverse compensation for example chemically inert, easy to modify, non-toxic, simple to assemble, steady in air and secure. Depending on the I-V technique theory, the current responses of 2-MNBBSH/GCE considerably changed during the adsorption of Y^3+^ by electrochemical approach. The probable mechanism towards 2-MNBBSH-Y complex formation is presented in Fig. 5. A similar interaction in perspective of yttrium complex formation has been reported in the previous studies^32^.

**Figure 6:**
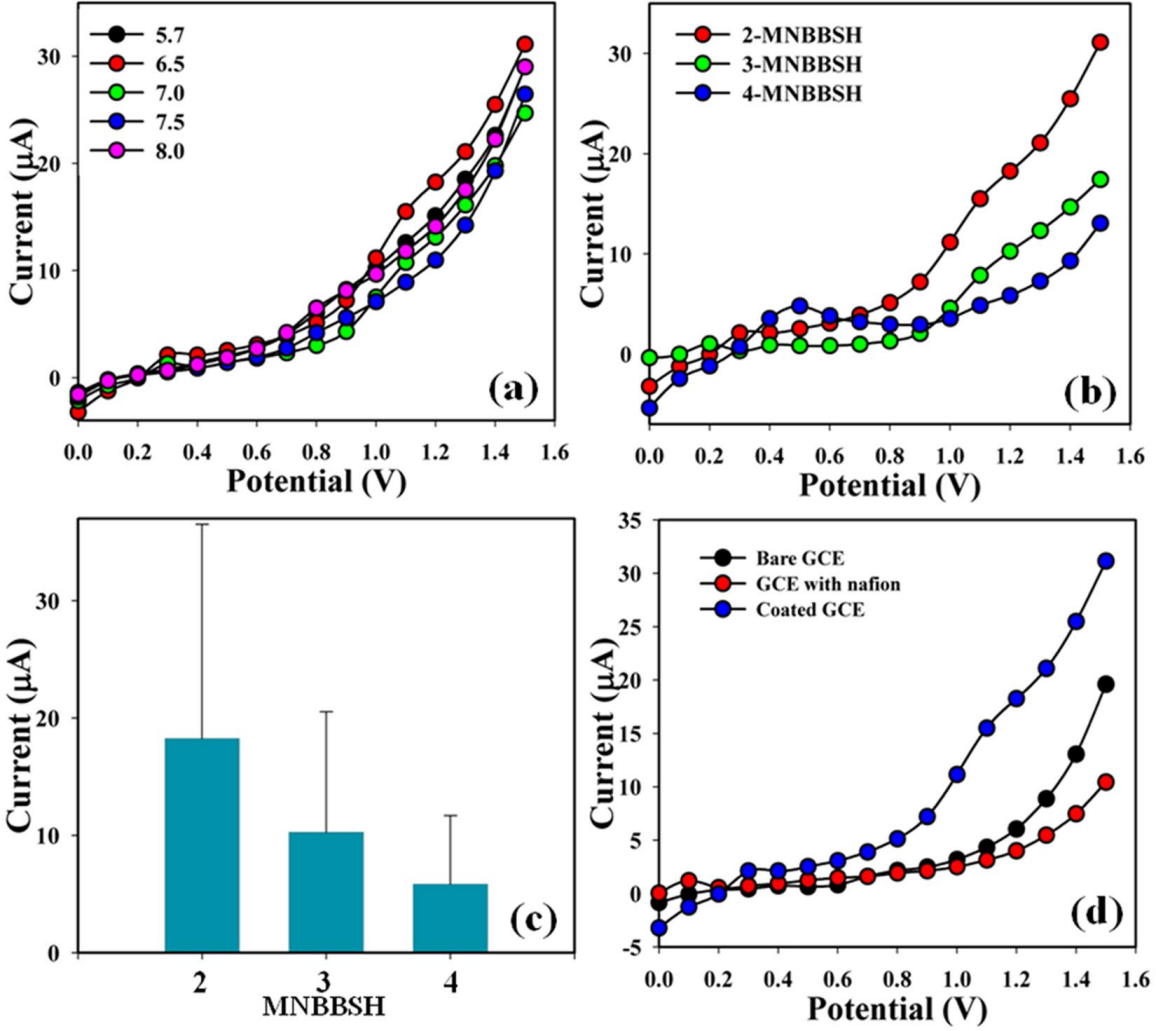
(**a**) pH optimization, (**b**) MNBBSH derivatives optimization in perspective of pH, (**c**) Bar diagram presentation of MNBBSH optimization at +1.2 V, and (**d**) Bare, and coated electrode.

The significant application of MNBBSH assembled onto an electrode as a MI sensor used for the detection of target cation that are less useful in biological and environmental arena. At first pH of the different PB was optimized in perspective of 2-MNBBSH to find out which PB system was more suitable to detect the desired MI, and pH = 6.5 showed more response compared with others towards the modified electrode (Fig. 6a). After that, derivatives of MNBBSH were optimized in PB (pH = 6.5) and 2-MNBBSH appeared additional responses (Fig. 6b). The current signals for the bare GCE, GCE with nafion and coated with 2-MNBBSH on the working electrode were presented in Fig. 6c. The differences of the current responses among bare, with nafion and coated GCE occurred due to current signals had been increased by coated in compared with bare and GCE with nafion.

**Figure 7:**
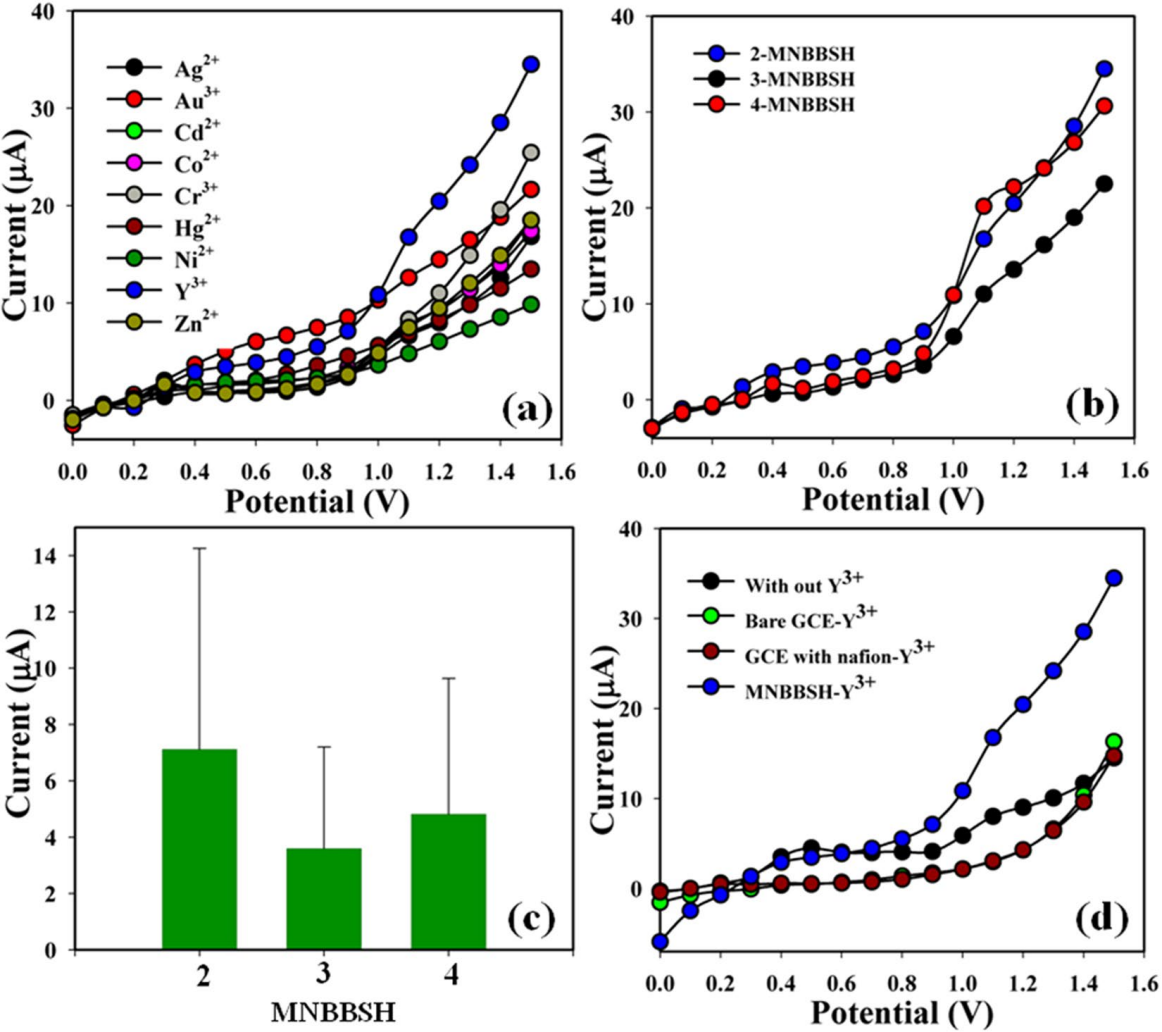
(**a**) Selectivity study, (**b**) Selectivity (Y^3+^) optimization in perspective of MNBBSH, (**c**) Bar-diagram presentation of selectivity optimization at +0.9 V, and (**d**) Absence and presence of Y^3+^ ions.

The metal ions such as Ag^2+^, Au^3+^, Cd^2+^, Co^2+^, Cr^3+^, Hg^2+^, Ni^2+^, Y^3+^, and Zn^2+^ were examined in order to find out the maximum current responses towards MNBBSH modified electrode, and consequently it was clearly observed that the sensor was more selective to Y^3+^ compared with other ions (Fig. 7a). The selectivity was optimized in perspective of MNBBSH derivatives and 2-MNBBSH appeared foremost response towards Y^3+^ (Fig. 7b). Figure 7c is the bar diagram presentation of selectivity optimization at +0.9 V. The current signal without metal ion (black-dotted) and with cation (green, brown, and blue dotted) were also performed (Fig. 7d). An increase of current responses observed regarding the modified 2-MNBBSH electrode with Y^3+^ which has given a large surface area with improved exposure in absorption, and adsorption potentiality onto the porous 2-MNBBSH surfaces of the target ion.

**Figure 8:**
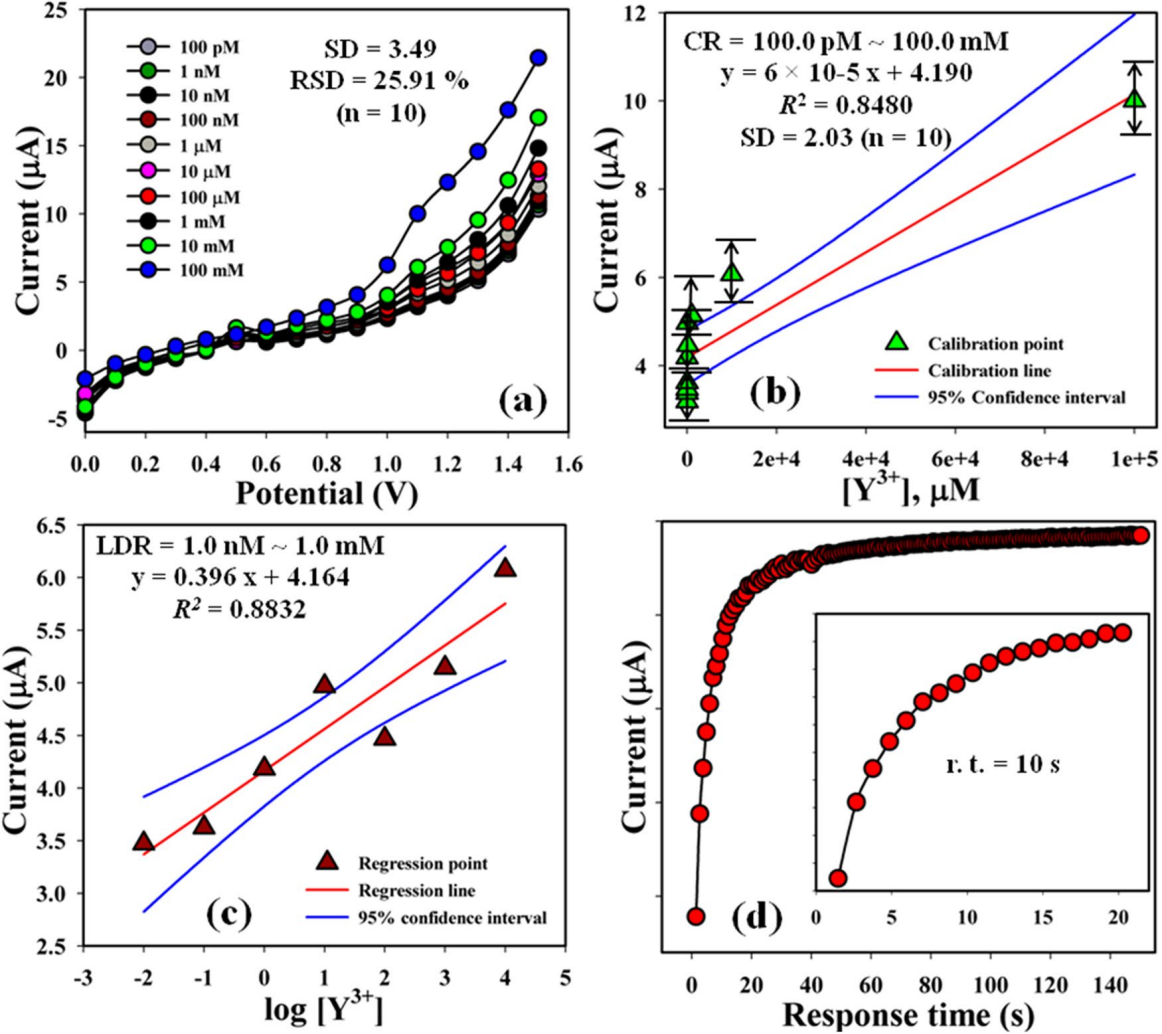
(**a**) Concentration variation, (**b**) Calibration curve at +1.1 V, (**c**) LDR graph, and (**d**) Response time.

The I-V responses of the Y^3+^ with diverse concentration (100.0 pM~100.0 mM) towards 2-MNBBSH modified electrode were investigated with indication of the changes of current of the fabricated electrode was a function of Y^3+^ concentration under normal condition, and it was reported that the current responses increased gradually from lower to higher concentration of the marked MI [SD = 3.49, RSD = 25.91% at +1.5 V, and n = 10] (Fig. 8a). A good range of the Y^3+^ concentrations were conducted from the lower to higher potential (0.0 ~ +1.5 V) in order to find out the feasible analytical limit. The linear calibration curve was plotted at +1.1 V from a range of Y^3+^ concentration (CR = 100.0 pM~100.0 mM, SD = 2.03, and n = 10), and found linear (*R* ^2^ = 0.8480) (Fig. 8b). Sensitivity (1.90 pAμM^−1^ cm^−2^), LOD (10.0 pM), and LOQ (33.33.mM) of the proposed modified electrode, 2-MNBBSH/GCE towards Y^3+^ were calculated from the calibration curve using the equation (i–iii), where m = slope of the calibration line, A = Active surface area of GCE, and SD = standard deviation of the calibration point. LDR (1.0 nM ~ 1.0 mM) was also calculated from the calibration graph and found linear, *R* ^2^ = 0.8832 (Fig. 8c). Response time (r. t.) of Y^3+^ towards 2-MNBBSH/GCE was measured at 1.0 μM and found 10.0 s (Fig. 8d).

i $${\boldsymbol{Sensitivity}}=\frac{{\boldsymbol{m}}}{{\boldsymbol{A}}}$$

ii $${\boldsymbol{LOD}}=\frac{({\bf{3}}\times {\boldsymbol{SD}})}{{\boldsymbol{m}}}$$

iii $${\boldsymbol{LOQ}}=\frac{({\bf{10}}\times {\boldsymbol{SD}})}{{\boldsymbol{m}}}$$

### Sensor stability examination

**Figure 9:**
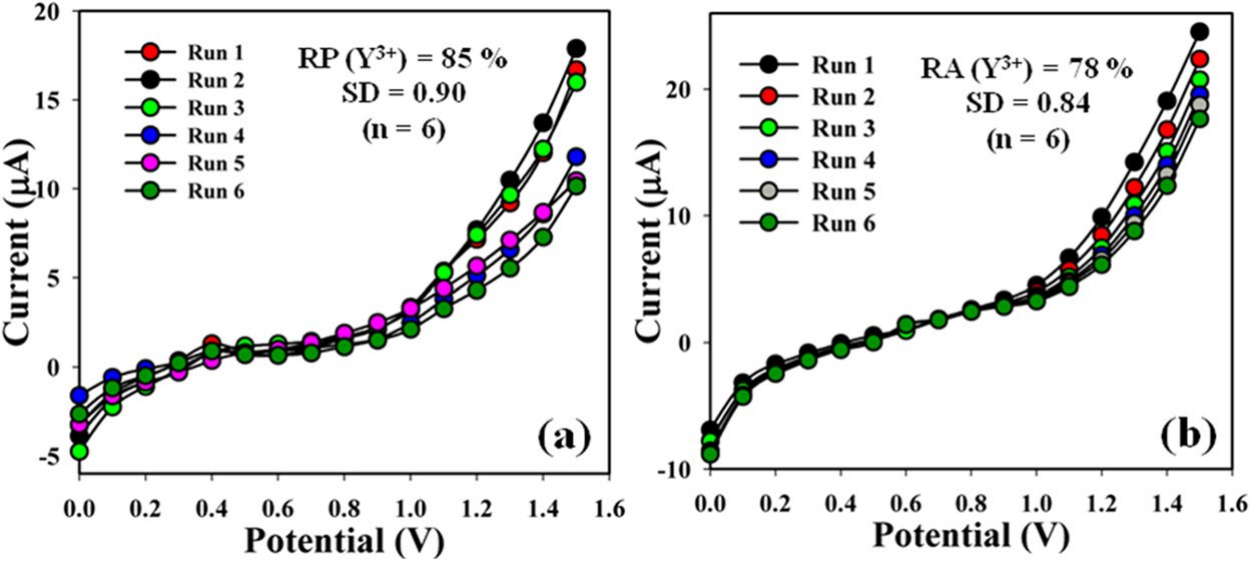
(**a**) Reproducible and (**b**) Repeatability study of 2-MNBBSH/GCE sensor.

The sensing ability of the 2-MNBBSH coated electrode was examined up to few weeks in order to the study of the reproducible (RP), and storage capabilities. Hence, a series of six successive magnitude of Y^3+^ solution (1.0 μM) was examined, and yielded outstanding reproducible responses with the 2-MNBBSH electrode at different environment (RP = 85%, SD = 0.90, and n = 6). It was recognized that the I-V responses were not comprehensively changes after washing of each experiment of the modified 2-MNBBSH electrode (Fig. 9a). The sensitivity remained similar around the original response up to two weeks, and after that the responses of the fabricated electrode become declined gradually. The responses of 2-MNBBSH sensor were considered with respect to storage time for the purpose of long term storage aptitude. The gratitude of storage ability of the 2-MNBBSH sensor was evaluated under regular array and the repeatability (RA) at calibrated potential (+1.1 V) was found 78% towards Y^3+^ for numerous days, SD = 0.84, and n = 6 (Fig. 9b, and Table S3). It was clearly reported that the modified sensor can be used without any considerable failure of sensitivity up to few weeks. A comparison of different MI detection using I-V method is presented in Table 3.

**Table 3:** Detection of various metal ions using I–V technique.

Modified electrodes	Metal ions	Sensitivity (μAμM^−1^ cm^−2^)	LOD (nM)	LDR (nM~mM)	Ref.
MPEBSH/GCE	Cd^2+^	2.22	0.14	0.35~350	33
EMDMBS/GCE	Co^2+^	1.87	0.17	0.35~3.5	34
TPCBZ/GCE	Cu^2+^	1.13	0.84	1.0~1.0	35
PEBSH/GCE	Hg^2+^	2.19	0.063	0.1~1.0	36
MNBBSH/GCE	Y^3+^	1.90 pAμM^−1^ cm^−2^	10.0 pM	1.0~1.0	This work

### Interference effect examination

**Figure 10:**
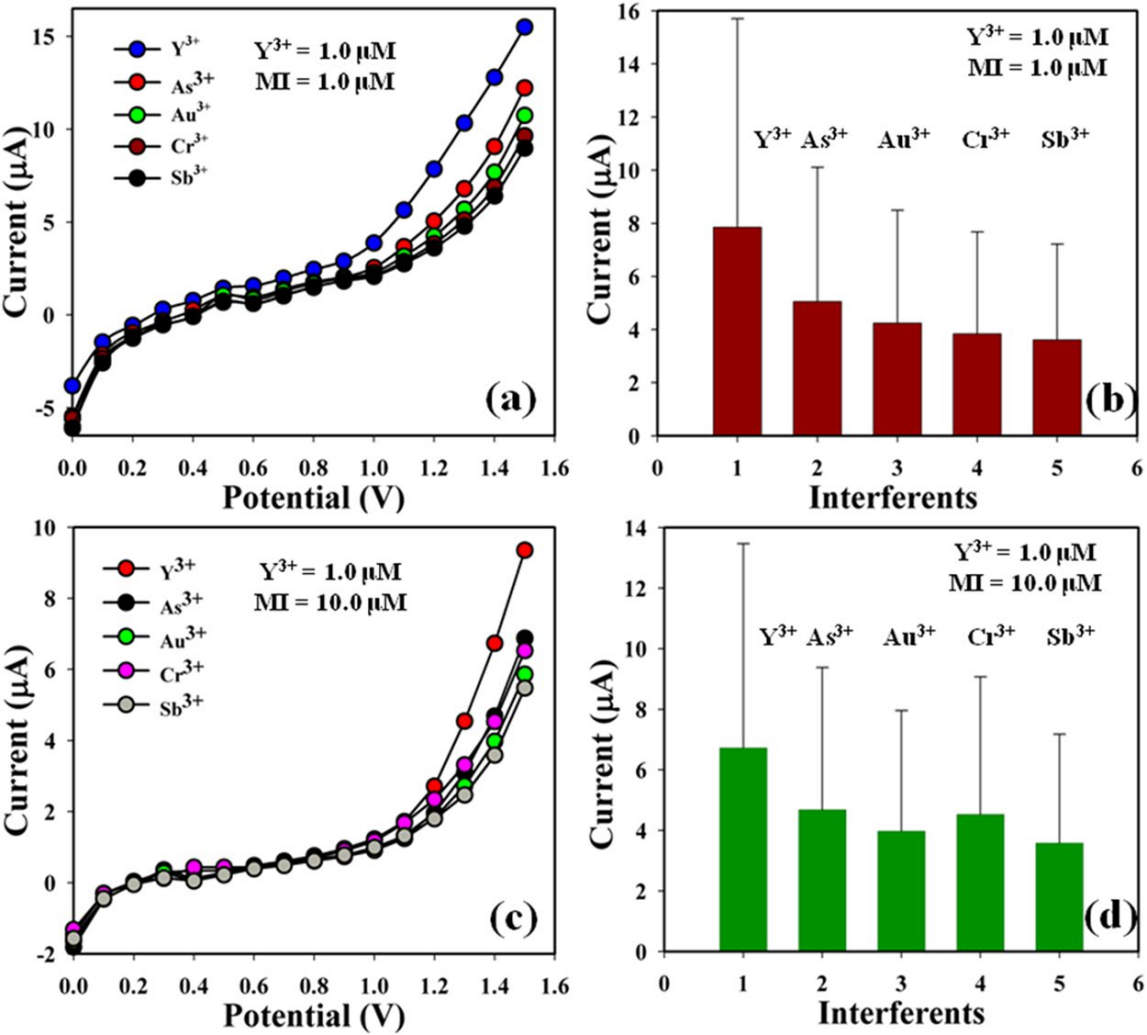
(**a**–**c**) Interference effect investigation and (**b**–**d**) bar diagram presentation of IE at +1.2, and +1.4 V respectively.

Examination of interference effect is one of the considerable practices in analytical science having the capability in order to make different the interfering agents from the MI bearing similar cationic nature. As^3+^, Au^3+^, Cr^3+^, and Sb^3+^ were usually used as interfering MI in the electrochemical Y^3+^ detection approach. I-V responses at 2-MNBBSH/GCE sensor toward the addition of Y^3+^ (1.0 μM), and interfering MI such as As^3+^, Au^3+^, Cr^3+^, and Sb^3+^ (1.0 and 10.0 μM, ~25.0 μL) in PB (pH = 6.5, and 10.0 mL) were investigated. The effects of interfering cations towards Y^3+^ were calculated from the experiential current (μA) at calibrated potential (+1.1 V), where the interfering effect of Y^3+^ was considered to be 100% (Fig. 10a–d, and Table S4). It was noticeable that 2-MNBBSH/GCE did not show any major current responses towards interfering MI. As a result, the expected sensor is proper for the finding of Y^3+^ with admirable sensitivity.

### Prospective analysis of real samples

**Figure 11:**
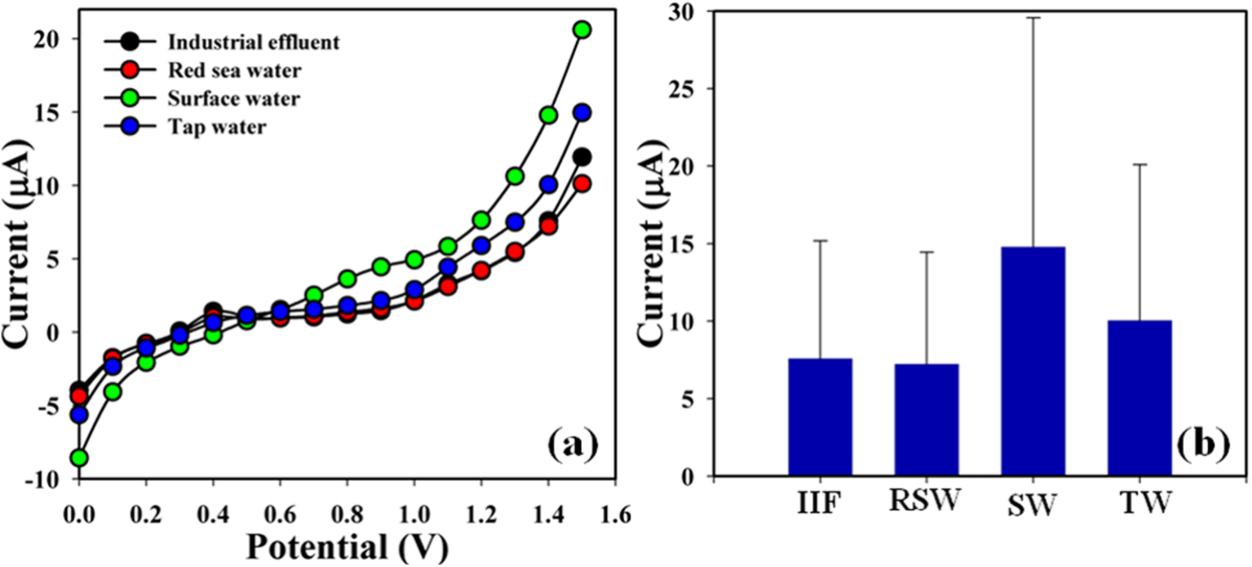
(**a**) Real samples analysis and (**b**) Bar diagram presentation of RS at +1.4 V.

The real samples (RS) such as industrial effluent (IE), red sea water (RSW), surface water (SW), and tap water (TW) were examined in order to endorse the expected I-V scheme using 2-MNBBSH/GCE. A usual addition method was used to measure the concentration of Y^3+^ in true samples. A fixed amount (~25.0 µL) of each RS was analyzed in PB (10.0 mL, 100.0 mM) using the fabricated 2-MNBBSH/GCE. The results were included at calibrated potential (+1.1 V) regarding the determination of Y^3+^ in IE, RSW, SW, and TW which truly confirmed the projected I-V procedure is suitable, dependable, and appropriate for analyzing of RS (Fig. 11a–b, and Table 4).

**Table 4:** Analysis of real samples by MNBBSH/GCE sensor.

RS	OC (μA)						Conc. (μM)	SD (n = 5)
	R1	R2	R3	R4	R5	Average		
IE	6.94	2.97	2.31	2.05	2.02	3.26	0.78	2.09
RSW	6.14	2.91	2.35	2.03	2.05	3.10	0.74	1.74
SW	6.24	7.41	5.51	5.12	4.89	5.83	1.39	1.02
TW	9.41	4.16	3.15	2.83	2.56	4.42	1.06	2.85

*RS: Real sample*, *IE: Industrial effluent*, *RSW*: *Red sea water*, *SW*: *Surface water*, *TW*: *Tap water*, *R*: *Reading*, *OC*: *Observed current*, *and SD*: *Standard deviation*.

## Conclusion

Three novel MNBBSH compounds were synthesized, characterized, and potentially applied in order to detect the toxic trivalent metallic cation using I-V performance. Systematic performances of trivalent Y^3+^ sensor using 2-MNBBSH/GCE were examined by a reliable I-V technique in terms of sensitivity, LOD, LOQ, LDR, response time, and reproducibility. This sensor advancement was confirmed to the higher selectivity and fast detection of Y^3+^ using 2-MNBBSH modified GCE with nafion matrix. An innovative development regarding MI detection can be introduced from this novel approach for monitoring of toxic cations in biological and environmental field.

## Electronic supplementary material

Supplementary file
