# Supplementary material for: Trivalent Y3+ ionic sensor development based on (E)-Methyl-N′-nitrobenzylidene-benzenesulfonohydrazide (MNBBSH) derivatives modified with nafion matrix
Source: Sci Rep. 2017 Jul 19;7:5832. doi: 10.1038/s41598-017-05703-4 (PMC5517609; doi:10.1038/s41598-017-05703-4)
Supplement: Supplementary file 1 — Supplementary file [file 41598_2017_5703_MOESM1_ESM.doc]

**Trivalent Y^3+^ ionic sensor development based on (*E*)-Methyl-*N*ʹ-nitrobenzylidene-benzenesulphonohydrazide (MNBBSH) derivatives modified with nafion matrix†**

**Mohammad Musarraf Hussain, Mohammed M. Rahman*, Muhammad Nadeem Arshad, Abdullah M. Asiri**

*Chemistry Department and Center of Excellence for Advanced Material Research, Faculty of Science, King Abdulaziz University, Jeddah 21589, P.O. Box 80203, Saudi Arabia*

**Electronic supplementary materials (ESM)**


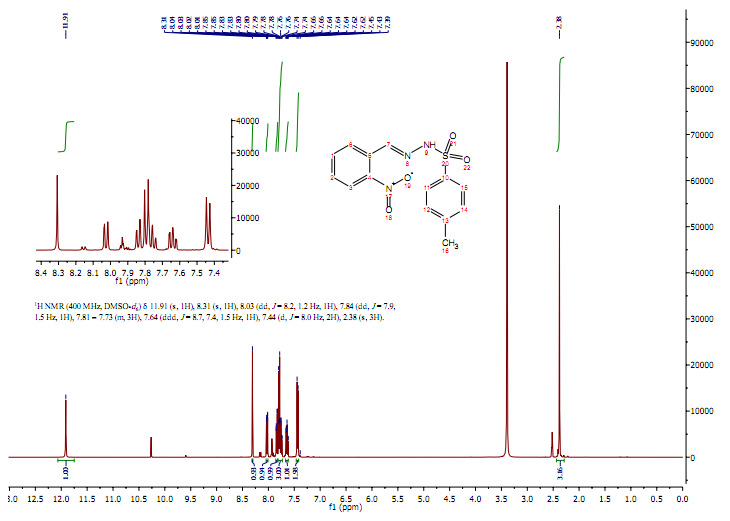


Figure S1: ^1^H-NMR of 2-MNBBSH.


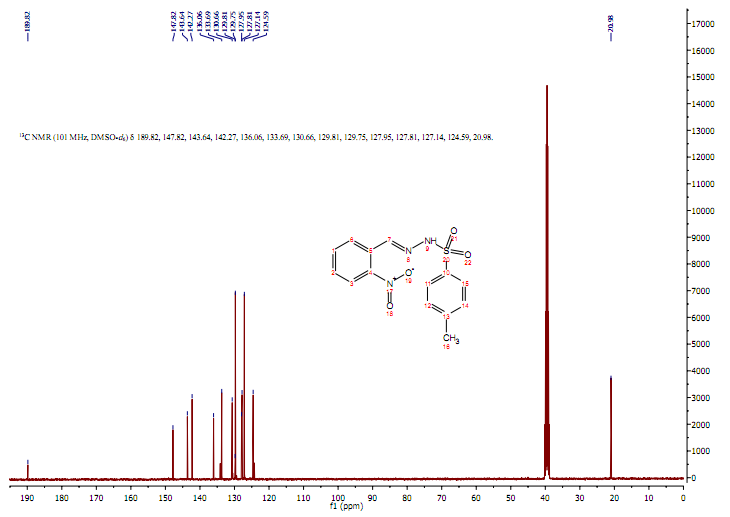


Figure S2: ^13^C-NMR of 2-MNBBSH.

**
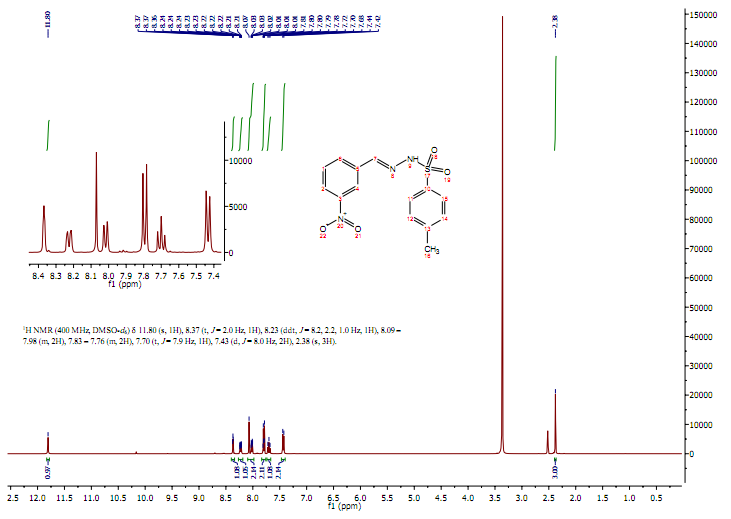
**

Figure S3: ^1^H-NMR of 3-MNBBSH.

**
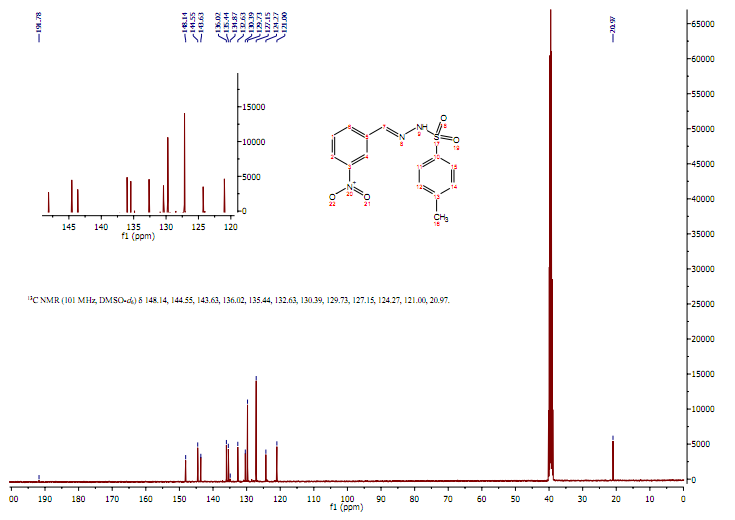
**

Figure S4: ^13^C-NMR of 3-MNBBSH.


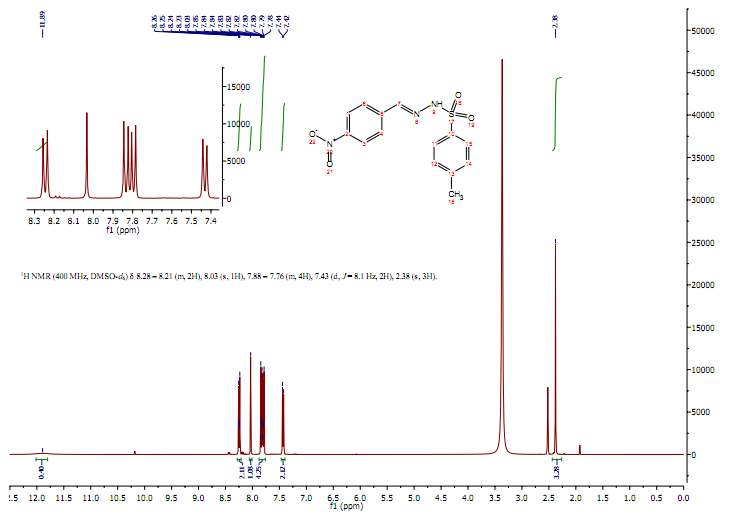


Figure S5: ^1^H-NMR of 4-MNBBSH.


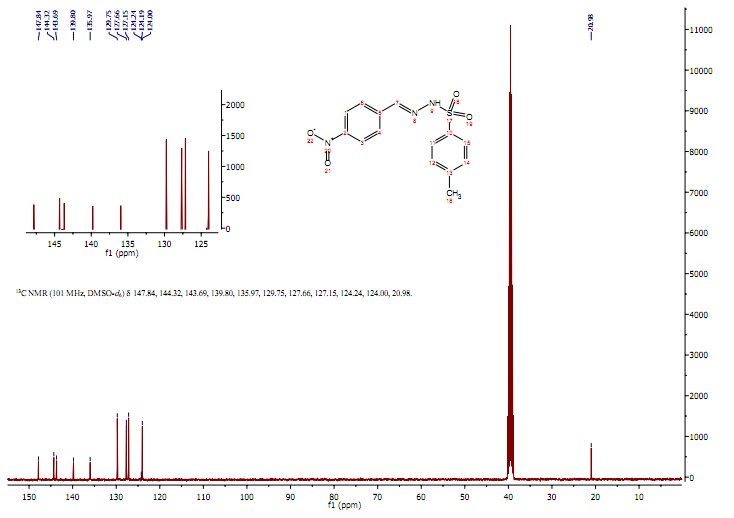


Figure S6: ^13^C-NMR of 4-MNBBSH.


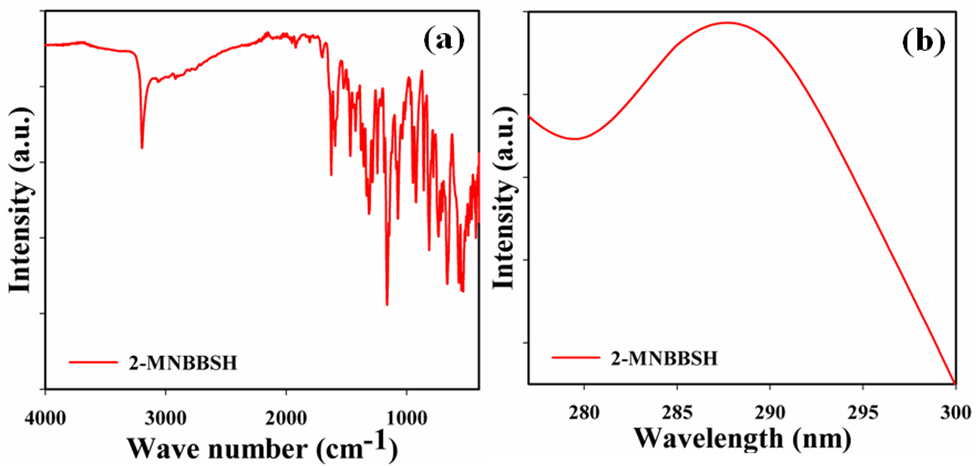


Figure S7: FTIR, and UV-Vis spectra of 2-MNBBSH.


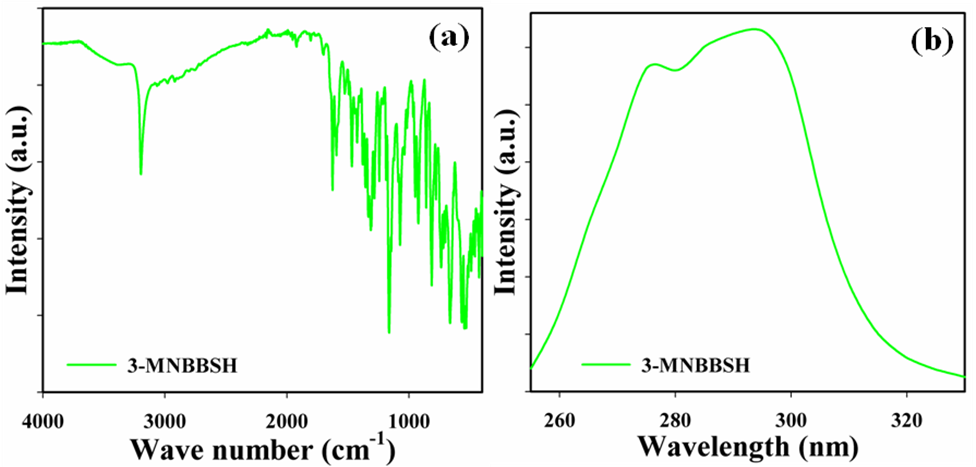


Figure S8: FTIR, and UV-Vis spectra of 3-MNBBSH.


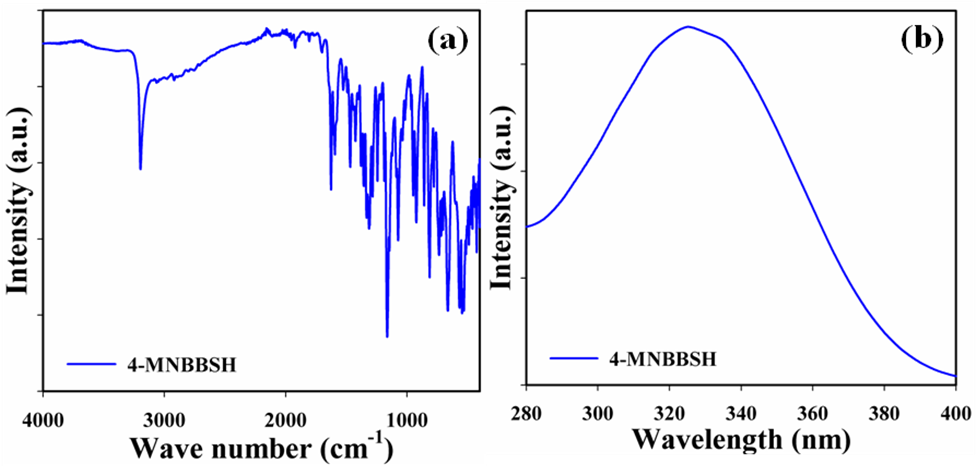


Figure S9: FTIR, and UV-Vis spectra of 4-MNBBSH.

**Table S1:** Bond angles for MNBBSH molecules (3-5).

| **2-MNBBSH (3)** | | | | | | | |
| --- | --- | --- | --- | --- | --- | --- | --- |
| **Atom** | **Atom** | **Atom** | **Angle/˚** | **Atom** | **Atom** | **Atom** | **Angle/˚** |
| O1 | S1 | N1 | 103.74(9) | C3 | C4 | C5 | 117.8(2) |
| O1 | S1 | C1 | 108.93(9) | C3 | C4 | C14 | 121.3(2) |
| O2 | S1 | O1 | 120.43(9) | C5 | C4 | C14 | 120.9(2) |
| O2 | S1 | N1 | 107.76(9) | C4 | C5 | C6 | 121.6(2) |
| O2 | S1 | C1 | 108.36(9) | C1 | C6 | C5 | 119.2(2) |
| N1 | S1 | C1 | 106.83(9) | N2 | C7 | C8 | 119.04(16) |
| N2 | N1 | S1 | 116.93(13) | C9 | C8 | C7 | 125.49(16) |
| C7 | N2 | N1 | 115.23(15) | C9 | C8 | C13 | 115.41(16) |
| O3 | N3 | O4 | 123.02(18) | C13 | C8 | C7 | 119.07(17) |
| O3 | N3 | C9 | 119.44(16) | C8 | C9 | N3 | 121.41(16) |
| O4 | N3 | C9 | 117.53(17) | C10 | C9 | N3 | 115.91(17) |
| C2 | C1 | S1 | 119.67(15) | C10 | C9 | C8 | 122.68(17) |
| C6 | C1 | S1 | 119.75(15) | C11 | C10 | C9 | 119.94(18) |
| C6 | C1 | C2 | 120.58(19) | C10 | C11 | C12 | 119.07(18) |
| C3 | C2 | C1 | 119.1(2) | C13 | C12 | C11 | 120.69(19) |
| C2 | C3 | C4 | 121.7(2) | C12 | C13 | C8 | 122.13(19) |
| **3-MNBBSH (4)** | | | | | | | |
| O1 | S1 | N1 | 103.85(19) | C3 | C4 | C14 | 121.7(4) |
| O1 | S1 | C1 | 108.94(17) | C5 | C4 | C3 | 118.2(3) |
| O2 | S1 | O1 | 120.2(2) | C5 | C4 | C14 | 120.1(4) |
| O2 | S1 | N1 | 107.4(2) | C4 | C5 | C6 | 121.5(4) |
| O2 | S1 | C1 | 107.2(2) | C1 | C6 | C5 | 119.0(4) |
| N1 | S1 | C1 | 108.79(18) | N2 | C7 | C8 | 120.1(3) |
| N2 | N1 | S1 | 115.5(3) | C9 | C8 | C7 | 119.9(3) |
| C7 | N2 | N1 | 115.8(3) | C9 | C8 | C13 | 118.4(4) |
| O3 | N3 | O4 | 123.5(4) | C13 | C8 | C7 | 121.6(3) |
| O3 | N3 | C10 | 117.2(4) | C8 | C9 | C10 | 119.7(3) |
| O4 | N3 | C10 | 119.3(4) | C9 | C10 | N3 | 119.5(3) |
| C2 | C1 | S1 | 120.7(3) | C11 | C10 | N3 | 118.6(4) |
| C2 | C1 | C6 | 120.5(3) | C11 | C10 | C9 | 121.9(4) |
| C6 | C1 | S1 | 118.8(3) | C10 | C11 | C12 | 118.5(4) |
| C3 | C2 | C1 | 119.1(4) | C11 | C12 | C13 | 120.6(4) |
| C2 | C3 | C4 | 121.7(4) | C12 | C13 | C8 | 120.8(4) |
| **4-MNBBSH (5)** | | | | | | | |
| O1 | S1 | O2 | 120.08(12) | N2 | C7 | C8 | 121.1(2) |
| O1 | S1 | N1 | 104.11(13) | C9 | C8 | C7 | 119.3(2) |
| O1 | S1 | C1 | 111.00(12) | C13 | C8 | C7 | 121.6(2) |
| O2 | S1 | N1 | 106.72(13) | C13 | C8 | C9 | 119.1(2) |
| O2 | S1 | C1 | 107.27(12) | C10 | C9 | C8 | 120.6(2) |
| N1 | S1 | C1 | 106.85(12) | C11 | C10 | C9 | 118.8(2) |
| O5 | S2 | O6 | 120.13(13) | C10 | C11 | N3 | 118.7(2) |
| O5 | S2 | N4 | 105.22(12) | C10 | C11 | C12 | 122.2(2) |
| O5 | S2 | C15 | 109.93(13) | C12 | C11 | N3 | 119.0(2) |
| O6 | S2 | N4 | 106.54(12) | C13 | C12 | C11 | 118.6(2) |
| O6 | S2 | C15 | 107.57(12) | C12 | C13 | C8 | 120.8(2) |
| N4 | S2 | C15 | 106.66(12) | C16 | C15 | S2 | 120.1(2) |
| N2 | N1 | S1 | 114.83(19) | C20 | C15 | S2 | 119.7(2) |
| C7 | N2 | N1 | 115.4(2) | C20 | C15 | C16 | 120.1(3) |
| O3 | N3 | O4 | 122.4(3) | C17 | C16 | C15 | 119.4(2) |
| O3 | N3 | C11 | 119.2(3) | C16 | C17 | C18 | 121.4(3) |
| O4 | N3 | C11 | 118.4(2) | C17 | C18 | C28 | 120.5(3) |
| N5 | N4 | S2 | 112.46(17) | C19 | C18 | C17 | 118.2(3) |
| C21 | N5 | N4 | 116.8(2) | C19 | C18 | C28 | 121.3(3) |
| O7 | N6 | C25 | 118.1(2) | C18 | C19 | C20 | 121.5(3) |
| O8 | N6 | O7 | 123.5(3) | C19 | C20 | C15 | 119.4(3) |
| O8 | N6 | C25 | 118.4(2) | N5 | C21 | C22 | 119.8(2) |
| C2 | C1 | S1 | 120.5(2) | C23 | C22 | C21 | 119.9(2) |
| C2 | C1 | C6 | 119.9(3) | C23 | C22 | C27 | 119.1(2) |
| C6 | C1 | S1 | 119.6(2) | C27 | C22 | C21 | 121.0(2) |
| C3 | C2 | C1 | 119.8(3) | C24 | C23 | C22 | 120.6(2) |
| C4 | C3 | C2 | 121.4(3) | C25 | C24 | C23 | 118.5(2) |
| C3 | C4 | C5 | 118.3(3) | C24 | C25 | N6 | 118.7(2) |
| C3 | C4 | C14 | 120.6(3) | C24 | C25 | C26 | 122.6(2) |
| C5 | C4 | C14 | 121.2(3) | C26 | C25 | N6 | 118.7(2) |
| C4 | C5 | C6 | 121.5(3) | C25 | C26 | C27 | 118.4(2) |
| C1 | C6 | C5 | 119.1(3) | C26 | C27 | C22 | 120.9(2) |

**Table S2:** Bond lengths for MNBBSH molecules (3-5).

| **2-MNBBSH (3)** | | | | | |
| --- | --- | --- | --- | --- | --- |
| **Atom** | **Atom** | **Length/Å** | **Atom** | **Atom** | **Length/Å** |
| S1 | O1 | 1.4247(14) | C3 | C4 | 1.382(3) |
| S1 | O2 | 1.4230(14) | C4 | C5 | 1.382(3) |
| S1 | N1 | 1.6377(17) | C4 | C14 | 1.508(3) |
| S1 | C1 | 1.7601(19) | C5 | C6 | 1.384(3) |
| O3 | N3 | 1.218(2) | C7 | C8 | 1.467(2) |
| O4 | N3 | 1.219(2) | C8 | C9 | 1.397(2) |
| N1 | N2 | 1.380(2) | C8 | C13 | 1.403(2) |
| N2 | C7 | 1.269(2) | C9 | C10 | 1.380(3) |
| N3 | C9 | 1.469(2) | C10 | C11 | 1.375(3) |
| C1 | C2 | 1.381(3) | C11 | C12 | 1.378(3) |
| C1 | C6 | 1.376(3) | C12 | C13 | 1.371(3) |
| C2 | C3 | 1.379(3) |  |  |  |
| **3-MNBBSH (4)** | | | | | |
| S1 | O1 | 1.430(3) | C3 | C4 | 1.377(6) |
| S1 | O2 | 1.421(3) | C4 | C5 | 1.374(6) |
| S1 | N1 | 1.626(4) | C4 | C14 | 1.515(6) |
| S1 | C1 | 1.754(4) | C5 | C6 | 1.383(5) |
| O3 | N3 | 1.201(5) | C7 | C8 | 1.469(5) |
| O4 | N3 | 1.203(5) | C8 | C9 | 1.381(5) |
| N1 | N2 | 1.396(4) | C8 | C13 | 1.392(5) |
| N2 | C7 | 1.259(5) | C9 | C10 | 1.382(5) |
| N3 | C10 | 1.458(5) | C10 | C11 | 1.370(6) |
| C1 | C2 | 1.378(5) | C11 | C12 | 1.375(6) |
| C1 | C6 | 1.380(5) | C12 | C13 | 1.377(6) |
| C2 | C3 | 1.376(5) |  |  |  |
| **4-MNBBSH (5)** | | | | | |
| S1 | O1 | 1.4224(18) | C4 | C14 | 1.515(4) |
| S1 | O2 | 1.4305(19) | C5 | C6 | 1.383(4) |
| S1 | N1 | 1.646(2) | C7 | C8 | 1.467(3) |
| S1 | C1 | 1.750(3) | C8 | C9 | 1.394(3) |
| S2 | O5 | 1.4223(19) | C8 | C13 | 1.384(3) |
| S2 | O6 | 1.4329(19) | C9 | C10 | 1.375(4) |
| S2 | N4 | 1.639(2) | C10 | C11 | 1.369(4) |
| S2 | C15 | 1.749(3) | C11 | C12 | 1.378(3) |
| O3 | N3 | 1.206(3) | C12 | C13 | 1.373(3) |
| O4 | N3 | 1.208(3) | C15 | C16 | 1.384(3) |
| O7 | N6 | 1.225(3) | C15 | C20 | 1.383(4) |
| O8 | N6 | 1.217(3) | C16 | C17 | 1.378(4) |
| N1 | N2 | 1.394(3) | C17 | C18 | 1.383(4) |
| N2 | C7 | 1.261(3) | C18 | C19 | 1.377(4) |
| N3 | C11 | 1.471(3) | C18 | C28 | 1.504(4) |
| N4 | N5 | 1.392(3) | C19 | C20 | 1.379(4) |
| N5 | C21 | 1.267(3) | C21 | C22 | 1.465(3) |
| N6 | C25 | 1.466(3) | C22 | C23 | 1.389(3) |
| C1 | C2 | 1.375(4) | C22 | C27 | 1.390(3) |
| C1 | C6 | 1.377(4) | C23 | C24 | 1.381(3) |
| C2 | C3 | 1.374(4) | C24 | C25 | 1.371(3) |
| C3 | C4 | 1.369(4) | C25 | C26 | 1.375(3) |
| C4 | C5 | 1.371(5) | C26 | C27 | 1.375(4) |

**Table S3:** Reproduce and repeatability study of the proposed 2-MNBBSH/GCE sensor.

| Run | Current (μA) | Reproducibility (%) | | Current (μA) | Repeatability (%) | |
| --- | --- | --- | --- | --- | --- | --- |
|  |  | Individual | Average |  | Individual | Average |
| 1 | 5.37 | 100 |  | 6.67 | 100 |  |
| 2 | 5.34 | 99 |  | 5.74 | 86 |  |
| 3 | 5.28 | 98 | 85 | 5.14 | 77 | 78 |
| 4 | 3.81 | 71 |  | 4.83 | 72 |  |
| 4 | 4.40 | 82 |  | 4.63 | 69 |  |
| 6 | 3.27 | 61 |  | 4.39 | 66 |  |

*Reproducibility and repeatability of run 1 have been considered as 100 %.*

**Table S4: Interference effect study of metal ions towards 2-MNBBSH/GCE sensor.**

| ME | MI | Conc. (μM) | Observed current (μA) | | | | IE (%) | SD (n = 3) |
| --- | --- | --- | --- | --- | --- | --- | --- | --- |
|  |  |  | R1 | R2 | R3 | Average |  |  |
|  | Y^3+^ | 1.0 | 7.74 | 5.13 | 4.08 | 5.65 | 100 | 1.88 |
|  | As^3+^ |  | 4.40 | 3.42 | 3.20 | 3.67 | 65 | 0.64 |
| 1 | Au^3+^ | 1.0 | 3.50 | 3.05 | 2.93 | 3.16 | 56 | 0.30 |
|  | Cr^3+^ |  | 3.16 | 2.86 | 2.60 | 2.87 | 51 | 0.28 |
|  | Sb^3+^ |  | 2.90 | 2.67 | 2.70 | 2.76 | 49 | 0.13 |
|  | Y^3+^ | 1.0 | 2.20 | 1.58 | 1.37 | 1.72 | 100 | 0.43 |
|  | As^3+^ |  | 1.45 | 1.28 | 1.25 | 1.33 | 77 | 0.11 |
| 2 | Au^3+^ | 10.0 | 1.30 | 1.20 | 1.23 | 1.24 | 72 | 0.05 |
|  | Cr^3+^ |  | 2.10 | 1.55 | 1.40 | 1.68 | 98 | 0.37 |
|  | Sb^3+^ |  | 1.41 | 1.29 | 1.25 | 1.32 | 77 | 0.08 |

*ME = Modified electrode, MI = Metal ions, R = Reading, IE = Interference effect, SD = Standard deviation, RSD = Relative standard deviation, and interference effect of Y^3+^ was considered to be 100 %.*
